# TPPA titer as a new adaptation for early diagnosis of congenital syphilis: a retrospective analysis of observation over three years in Yunnan, China

**DOI:** 10.1186/s40001-019-0367-8

**Published:** 2019-02-02

**Authors:** Hong-Mei Wang, Yu-Ye Li, Li-Ping He, Ying-Kui Cao, Tian-Xiang Dong, Yi-Qun Kuang

**Affiliations:** 1 Department of Dermatology and Venereology, First Affiliated Hospital of Kunming Medical University, Kunming, 650032 China; 2 Institute of Infection and Immunity, Henan University & Center for Translational Medicine, Huaihe Clinical College, Huaihe Hospital of Henan University, Kaifeng, 475000 China; 3 School of Public Health, Kunming Medical University, Kunming, 650500 China

**Keywords:** Congenital syphilis, Passive antibodies, Seroreversion discipline, Early diagnosis

## Abstract

**Background:**

Early diagnosis of congenital syphilis (CS) is difficult. This study aimed to determine the serological response discipline of syphilis passive antibody (SPA) in infants born to mothers with syphilis and provide the basis for the early diagnosis of CS.

**Methods:**

Thirty-three infants born to mothers with syphilis and six infants with CS were recruited. The toluidine red unheated serum test (TRUST) and *Treponema pallidum* particle agglutination (TPPA) titers were followed up after birth.

**Results:**

The results showed that serological response in the serum of infants with the TPPA titer decreased threefold at 3rd month, or the titer dropped to a minimum of 1:40 at 9th month was SPA. The TPPA titer of 6 CS cases remained positive after 3-year follow-up, and the titer did not decline after treatment and maintained longer. The infants with TRUST titer ≥ 1:4 at birth were prone to become syphilis serofast, while TRUST titer < 1:4 turned to negative quickly.

**Conclusion:**

In infants with SPA, the TRUST converted negative earlier than the TPPA. The lower the TPPA initial titer was, the shorter the seroreversion time required. The TPPA titer can be used to predict CS in infants born to mothers with syphilis.

## Background

Congenital syphilis (CS) is caused by *Treponema pallidum* through the maternal placenta, amniotic tissue or blood circulation to infect the fetus [1]. The diagnosis of CS without clinical symptoms is a worldwide challenge. Currently, it is mainly relied on serological tests, including non-*Treponema pallidum* serum test such as toluidine red unheated serum test (TRUST), as well as the *Treponema pallidum* serum test such as *Treponema pallidum* particle agglutination (TPPA). Neonates with serologically positive by TRUST and TPPA tests cannot be clearly diagnosed as CS, because the non-*Treponema pallidum* or *Treponema pallidum* IgG antibody of mother can be transferred to the fetus [2]. These antibodies are called syphilis passive antibodies (SPA). Strict follow-up of newborns or infants produced by women with syphilis is a necessary means for diagnosis of latent CS. Infants born to pregnant women with syphilis are followed up to 18 months; only the *Treponema pallidum* serology test maintaining positive is diagnosed as CS [3]. Mucocutaneous manifestations are presented in about 70% of infants with early CS [4], and it is classically a vesiculobullous or maculopapular rash occurring on the palms and soles of the infants [4, 5]; other signs like premature delivery, low birth weight, hepatosplenomegaly, pneumonitis, etc. have been observed [6]. However, CS is often manifested as latent syphilis, about 60% infants at birth without clinical symptoms, which results in a certain difficulty in early diagnosis. The diagnosis of asymptomatic CS was based on laboratory findings as a basis, for follow-up of 18-month TPPA positive as a diagnostic standard for CS. Due to the long follow-up time required by the traditional diagnosis of CS, it results in high rate of loss to follow-up, and makes the resource of stress to the family. Here, we carried out a follow-up study with TPPA and TRUST tests on in infants born to mother with syphilis, aiming to study the seroreversion discipline, thus providing evidence for the possibility of immediate early diagnosis of CS.

## Patients and methods

### Ethics

This study was approved by the ethics review boards of Kunming Medical University and Henan University. The written informed consent was obtained from the study participants; parental consent was obtained for participated infants. All experiments were performed in accordance with the approved guidelines and regulations according to the principles expressed in the Declaration of Helsinki, and the experimental protocols were approved by the institutional review boards of the universities.

### Subjects

The participants were outpatients (follow-up pregnant women with syphilis and their infants) at the dermatology and venereology clinic in the First Affiliated Hospital of Kunming Medical University from January 2010 to December 2016. The diagnosis of pregnancy syphilis and CS, syphilis staging, and treatment standards are based on the United States guidelines [3].

The laboratory diagnostic criteria of CS used in this study were infants with TPPA continued to be positive at 18th month after birth. The SPA group was infants who had complete dynamic TPPA and TRUST testing data, and the TPPA titer converted negative at 18th month after birth. CS group was infants whose TPPA maintained positive over 18-month follow-up after birth.

### TPPA and TRUST tests

The venous blood of pregnant women with syphilis and corresponding infants was collected, and then subjected to TPPA test and TRUST titer test (Fuji Rimini Co. Ltd). The treatment regimens, follow-up time, and serum titer were recorded. The TPPA and TRUST titers of infants were measured at the initial visit, 1, 3, 5, 9, 12, 15, 18 months after birth. The TPPA and TRUST titers of the syphilis-positive women were measured at the times before and after treatment, the first visit during pregnancy, and delivery.

### Treatment regimens

Intramuscular benzathine penicillin G (BPG) was applied as the first choice to pregnant women with syphilis: 2.4 millions U of BPG once weekly for 3 consecutive weeks. Each anti-syphilis regimen course was carried, respectively, at first 3 months of pregnancy and the last 3 months of pregnancy. The regimen for the infants with CS was once intragluteal injection of 50,000 U/kg/day of BPG. The patients allergic to BPG were replaced by 250 mg/day of ceftriaxone intramuscularly; this regimen was continued for 10–14 days.

### Efficacy of treatment

TRUST converted negative or the titer declined by fourfold at 3rd month after treatment was defined as effective treatment. Cure was defined according to the criteria: early syphilis TRUST titer converted negative within 2 years (primary syphilis converted negative within 1 year, secondary syphilis converted negative within 2 years) or late syphilis (tertiary syphilis) converted negative within 3 years. The serofast syphilis was defined according to the criteria: TRUST remained positive and at a low titer after 2-year treatment in early syphilis, or in late syphilis for more than 2 years after treatment. Sero-relapse was defined if seroresponse turned positive shortly after a temporary negative conversion, or a fourfold increase in TRUST titer (such as 1:2 to 1:8).

### Statistical analysis

SPSS17.0 software was used for statistical analysis. The TPPA and TRUST titers were expressed by the median and interquartile range, and were subjected to the rank sum test of randomized block design. Value of *P* < 0.05 was statistically significant. The receiver operating characteristic (ROC) curve was employed to analyze the sensitivity and specificity of TPPA titer associated with congenital syphilis.

## Results

### Pregnant women with syphilis

A total of 31 pregnant women with syphilis whose infants with SPA (two of whom were mothers of the twin infants), and five women whose infants with CS were collected. SPA-positive infants’ mothers aged 19–38 years; CS infants’ mothers aged 20–27 years. Among 31 pregnant women with syphilis whose infants were with SPA, 29 cases (93.5%) were latent syphilis, and two cases (6.5%) were secondary syphilis, otherwise one case was co-infected with genital herpes. In contrast, all the mothers of CS infants were of latent syphilis. All women were HIV negative. Of the mothers of SPA infants, seven cases (22.6%) were found in pregnancy test, 11 cases (35.5%) were found in pre-pregnancy check, 12 cases (38.7%) were found during the physical examination, and one case of premarital examination (3.2%); seven cases (22.6%) were cured before pregnancy. Four cases (12.9%) were effective, six cases (19.4%) developed serofast, and seven cases were found before pregnancy and maintained the original titer after treatment (45.2%). There were seven cases (22.6%) identified by the pregnancy test, and the original titration maintained even under sustained treatment. For the CS infants’ mothers, one case of pre-pregnancy was identified by physical examination and displayed syphilis serum recurrence after treatment. Among four cases of syphilis identified during pregnancy screening, two cases demonstrated treatment effective, and the other two cases maintained the original titer. All the pregnant women with syphilis were treated with BPG.

### Infants with SPA or CS

There were 33 infants with SPA, all were full-term newborns, 15 cases were boys, and 18 cases were girls. The 33 SPA infants were TPPA positive at the time of birth; 4 of 33 cases were also TRUST positive, and no clinical symptoms were observed. There were six infants which were CS cases (Table 1).

**Table 1:** The clinical features of the six infants with congenital syphilis and corresponding mothers

Number	At birth		TRUST titer		TPPA titer		TRUST seroreversion time (M)
	Gestational age (W)	Weight (kg)	Infant	Mother	Infant	Mother	
1^a^	NA	NA	NA (0 Y)/1:64 (4 years)^b^	NA	NA (0 Y)/1:1280 (4 years)	NA	None^c^
2	38	3.14	1:1 (0 Y)/1:1 (1.5 years)	1:1	1:1280 (0 Y)/1:160 (1.5 years)	1:1280	30
3	32	1.83	1:8 (0 Y)/1:4 (4 years)	1:2	1:1280 (0 Y)/1:1280 (4 years)	1:1280	None
4	36	2.60	1:1 (0 Y)/− (11 years)	1:1	1:1280 (0 Y)/1:1280 (11 years)	1:1280	19
5	39	3.06	1:2 (0 Y)/− (4 years)	1:1	1:1280 (0 Y)/1:160 (4 years)	1:1280	6
6	38	2.92	1:8 (0 Y)/1:4 (5 years)	1:1	1:1280 (0 Y)/1:1280 (5 years)	1:1280	None

NA: not available; W: week(s); M: month(s); −: negative

^a^This infant is an adopted child, the information of the natural mother is not clear

^b^0 Y indicates the time of the infant at birth

^c^None means there is no seroreversion observed in the child

Of the CS infants, one case was an adoptive child whose information of birth and her natural mother were unknown, two cases were premature children, and three cases were full-term children. The first visit ages of the infants were 1.5–11 years, five girls and one boy; all infants were of latent syphilis. There were two infants born with a fourfold TRUST titer over the mother’s; they were treated with BPG and the TRUST did not convert negative under a 3-year follow-up. There were three infants born with less than fourfold TRUST titer of the mother’s; the TRUST converted negative rapidly. The one adoptive child was allergic to BPG and was treated with ceftriaxone sodium. The TRUST did not convert negative after 3-year follow-up (Table 1).

### The dynamics of TPPA titer in infants

Among the 33 infants with SPA, the TPPA titer negative conversion rate was 15.2%, 27.3%, 54.6%, 63.6%, 72.7% and 100% at the age of 3, 6, 9, 12, 15 and 18 months, respectively (Table 2). The TPPA titer of 33 SPA cases declined fast in first 3 months (decline by threefold), the titer reduced to the lowest level of 1:40 at 9th month and completely converted negative within 18 months (Table 3).

**Table 2:** The number of children with syphilis passive antibody at different seroreversion time points

	Total number of cases	Number of cases at seroreversion time point (n, %)					
		3 months	6 months	9 months	12 months	15 months	18 months
TRUST	4	0 (0)	1 (25)	2 (50)	1 (25)	0 (0)	0 (0)
TPPA	33	5 (15.2)	4 (12.1)	9 (27.3)	3 (9.1)	3 (9.1)	9 (27.3)

**Table 3:** TPPA titer in infants at different follow-up time points

Case number	TPPA titer (median [P25, P75]) at follow-up time point									*Z* value	*P* value
	First visit	3 M	6 M	9 M	12 M	15 M	18 M	24 M	36 M		
SPA											
33	480 [80, 1280]	160 [80, 320]	80 [0, 160]	40 [0, 80]	40 [20, 80]	40 [0, 80]	0	0	0	44.935	< 0.001
CS											
6	1280 [160–1280]	ND	ND	ND	960 [160–1280]	ND	ND	960 [160–1280]	960 [160–1280]	9.714	0.046

*M* month(s), *ND* not determined

The six infants with CS were followed up and checked the TPPA titer once each year for 3 years. The TPPA titer did not decline and maintained at the same titer for 3 years unchanged (Table 3). According to the rank sum test of random block designed data, there were significant differences in TPPA titers in the infants with SPA at initial visit, 3, 6, 9, 12, 15, and 18 months of age (*Z* = 44.935, *P* < 0.001). There were significant differences in TPPA titers among the CS infants at initial visit, 1, 2, and 3 years of age (*Z* = 9.714, *P* = 0.046).

### The dynamics of TRUST titer in infants

There were four infants with SPA who were TRUST positive. The initial TRUST titers were low, and began to convert negative at 6th month, and then completely converted negative at 12th month (Table 4).

**Table 4:** TRUST titer in infants at different follow-up time points

	Case	TRUST titer at follow-up time point						
		First visit	3 months	6 months	9 months	12 months	24 months	36 months
SPA	1	1:1	1:1	−	−	−	−	−
	2	1:4	1:2	1:1	1:1	−	−	−
	3	1:1	1:1	1:1	−	−	−	−
	4	1:1	1:1	1:1	−	−	−	−
CS	1	1:64	1:16	1:16	1:8	1:8	1:4	1:4
	2	1:1	1:1	1:1	1:1	−	−	−
	3	1:4	1:4	1:4	1:2	1:2	1:1	1:1
	4	−	−	−	−	−	−	−
	5	−	−	−	−	−	−	−
	6	1:4	1:4	1:4	1:4	1:4	1:4	1:2

−: negative

Of six infants with CS, three cases had a TRUST titer ≥ 1:4 at birth; the TRUST declined more slowly after treatment and showed serofast at last. In contrast, the other three cases had a titer < 1:4 at birth; the TRUST titer declined rapidly (Table 4).

### ROC curve estimation of predictive factor of CS

**Fig. 1:**
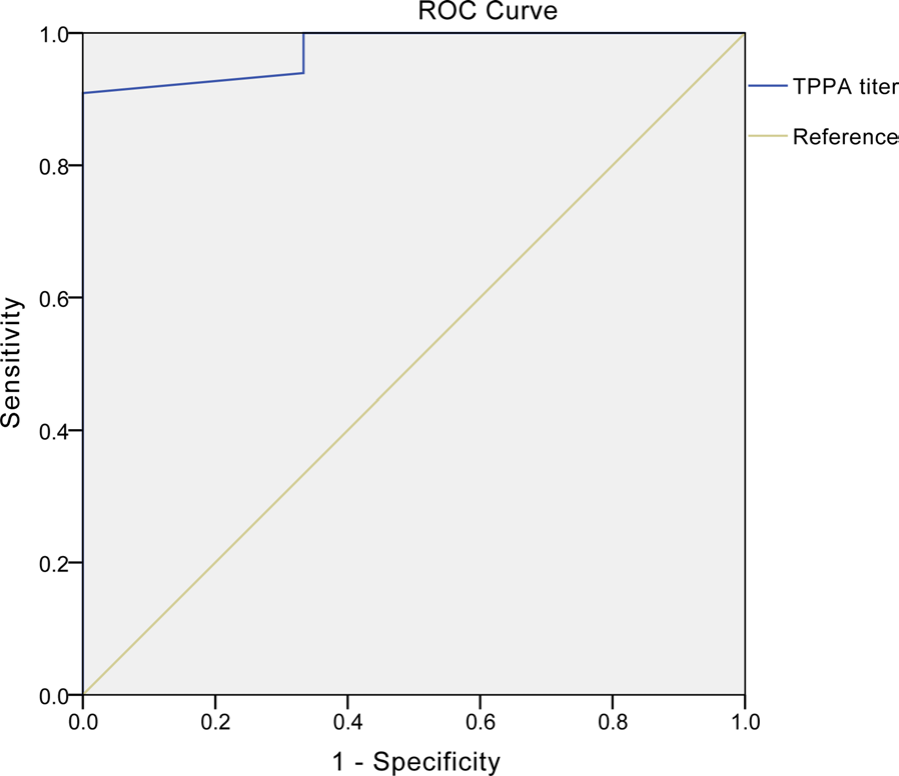
The ROC cure of predictive index. The *X*-axis depicts the specificity of predictor; the *Y*-axis indicates the sensitivity of the predictor. The diagonal line is the reference

Thirty-nine infants who had been followed up for 36 months were analyzed by ROC curve to find out the early predictive index of congenital syphilis. The result showed that TPPA titer at the end of the follow-up for 9 months had diagnostic value for SPA (Fig. 1). The area under the curve was 0.975 (*P* < 0.001, 95% CI (confidence interval) 0.001–0.1). The best diagnostic cut-off point of baseline TPPA titer was 1:400, the sensitivity was 93.9%, and the specificity was 66.7%. The false-positive rate was 33.3%, the false-negative rate was 6.1%; the positive predictive value was 33.9%, and the negative predictive value was 98.4%.

## Discussion

The TPPA monitoring among syphilis and HIV-1 co-infected patients had shown significance in treatment and prognosis [7]. Maternal and congenital syphilis decreased worldwide from 2008 to 2012 [1]. However, according to the data of Center for Diseases Control and Prevention of Yunnan Province, China, there were 53,559 cases of syphilis in Yunnan Province from 2011 to 2015, including 45,491 cases of latent syphilis, 8068 cases of primary, secondary, tertiary and congenital syphilis, of which 5764 cases (71.44%) were primary syphilis and 1799 cases (22.30%) were secondary syphilis, 139 cases (1.72%) of tertiary syphilis and 366 cases (4.54%) of congenital syphilis. Congenital syphilis accounts for such a high proportion, which may be partly due to misreporting of congenital syphilis in children with passive syphilis antibodies [8]. In this study, a strict follow-up over 3 years and detailed serological tests were conducted in infants with SPA and infants with CS. Especially, we analyze the dynamics of TPPA titer in infants born to mother with syphilis, which is the first exploration worldwide to our knowledge. Among the infants with SPA, the TPPA titer decreased rapidly (by threefold) in 3 months after birth. As of 9 months, it reduced to a minimum titer of 1:40 and completely converted negative at 18th months. While infants with CS had higher serum TPPA titers at births, the titer did not decline and maintained for many years at the same level even after sustained treatment. It indicates that according to the TPPA titer at the 3rd or 9th month in the infants born from pregnant women with syphilis, it could be used as early diagnosis of CS or SPA, which significantly reduced the diagnosis time of syphilis (18 months).

In this study, the TRUST in infants with SPA seroreverted within 12 months, while TPPA converted negative within 18 months. It has been reported early that the passively transferred maternal non-treponemal antibody titer usually declines in 3 months, and becomes negative in 6 months in non-infected infants. However, the TPPA in CS can persist for 12–15 months even under treatment [9]. The TPPA cumulative seroreversion rate was 63.64% and 100% at 12 and 18 months of age, respectively. The TRUST seroreversion time was significantly earlier than TPPA. It suggests that the lower the initial TPPA titer was, the shorter the time required for the seroreversion. This may be associated with the metabolism of the SPA in infants. The higher the concentration of SPA, the longer the metabolic time required. Likewise, the lower the concentration of SPA, the shorter the time of TPPA turns negative required. The results imply that the anti-syphilis treatment interventions with different regimens could prevent the CS [10, 11].

Pregnancy syphilis does not have clinical symptoms; 93.5% of the pregnant women with syphilis were latent syphilis in this study, which is higher than the Jewish population in Israeli (83.6%) reported in 2012 [12]. The mothers of five infants with CS were latent syphilis. Among them, one case displayed syphilis serofast after pre-pregnancy treatment; four cases that were diagnosed during pregnancy displayed stable original syphilis serum maintenance after treatment. There is controversy on pregnant women with syphilitic serofast will lead to CS, although adequate anti-syphilis treatment is critical for prevention of CS [13].

We employed the ROC curve analysis to evaluate the predictive value of TPPA titer in early diagnosis of CS. Our data showed high predictive sensitivity and specificity by the TPPA titer at 9th month after birth (Fig. 1). It has been reported that the classical reverse algorithm and two European Union algorithms evaluating EIA (enzyme immunoassay) or CLIA (chemiluminescence assay) could predict confirmatory TPPA results [14], but the TTPA titer was not tested. The reverse sequence screening algorithms on nine serological assays for diagnosis of syphilis also indicate an initial treponemal immunoassay screening subsequently followed by a second TrepSure test or TP-PA assay [15]. These algorithms suggested that multi-assay-based diagnosis of syphilis is labor-intensive and costless. Moreover, although a comparative Western Blot assay combining IgG and IgM has been shown to be allowing adequate identification of infants with CS [16], this test might be affected by the presence of antibodies produced by the neonate against other pathogens and potentially cross-reacting with *T. pallidum* antigens.

Although the ROC curve demonstrates a good predictive efficacy of early CS diagnosis by TPPA titer, our study had several limitations. The small sample sizes in the group SPA limited the statistical power for proving this conclusion, though the long-term strict follow-up of the cohorts demonstrated important information in the early diagnosis of CS. This study calls for a further investigation involving a larger number of mother–infant pairs in the future. The early diagnosis of CS is rapid and relatively cost-effective, if the clinicians could judge the infant’s syphilis status according to the TPPA titer at 9th month after birth, instead of conventional 18-month follow-up. CS is a preventable disease, which can be eliminated through effective antenatal screening and treatment of seropositive pregnant women [17, 18]. Early diagnosis of CS is the necessary prerequisite for timely treatment. Antenatal care interventions for syphilis in the first two trimesters of pregnancy can significantly reduce the risk of having an adverse outcome due to maternal syphilis [19, 20]. These findings are expected to be used as evidence for guiding our practice to prevent mother-to-child transmission of syphilis.

## Conclusions

In summary, SPA infants’ TRUST-negative rate was of 100% within 12 months, while TPPA seroreversion required 18 months. The lower the TPPA initial titer was, the shorter the seroreversion time required. Infants with the TPPA titer decreased threefold at the 3rd month after birth, or the TPPA titer dropped to a minimum titer of 1:40 at the 9th month after birth, can be suggested as SPA.

## Abbreviations

CS: congenital syphilis
SPA: syphilis passive antibody
TRUST: toluidine red unheated serum test
TPPA: *Treponema pallidum* particle agglutination
BPG: benzathine penicillin G
HIV: human immunodeficiency virus
ROC: receiver operating characteristic
EIA: enzyme immunoassay
CLIA: chemiluminescence assay
